# Tung Oil-Based Production of High 3-Hydroxyhexanoate-Containing Terpolymer Poly(3-Hydroxybutyrate-co-3-Hydroxyvalerate-co-3-Hydroxyhexanoate) Using Engineered *Ralstonia eutropha*

**DOI:** 10.3390/polym13071084

**Published:** 2021-03-29

**Authors:** Hye Soo Lee, Sun Mi Lee, Sol Lee Park, Tae-Rim Choi, Hun-Suk Song, Hyun-Joong Kim, Shashi Kant Bhatia, Ranjit Gurav, Yun-Gon Kim, June-Hyung Kim, Kwon-Young Choi, Yung-Hun Yang

**Affiliations:** 1 Department of Biological Engineering, College of Engineering, Konkuk University, Seoul 05029, Korea; lhs2265696@naver.com (H.S.L.); dltjsal6845@naver.com (S.M.L.); shckd2020@naver.com (S.L.P.); srim1004@gmail.com (T.-R.C.); shs9736@naver.com (H.-S.S.); sopero2@naver.com (H.-J.K.); shashibiotechhpu@gmail.com (S.K.B.); rnjtgurav@gmail.com (R.G.); 2 Institute for Ubiquitous Information Technology and Applications, Konkuk University, Seoul 05029, Korea; 3 Department of Chemical Engineering, Soongsil University, Seoul 06975, Korea; yungon.kim@gmail.com; 4 Department of Chemical Engineering, Dong-A University, Busan 49315, Korea; june0302@dau.ac.kr; 5 Department of Environmental and Safety Engineering, College of Engineering, Ajou University, Suwon 16499, Korea; kychoi@ajou.ac.kr

**Keywords:** tung oil, α-eleostearic acid, *Ralstonia eutropha*, polyhydroxyalkanoates, antioxidant, PHA

## Abstract

Polyhydroxyalkanoates (PHAs) are attractive new bioplastics for the replacement of plastics derived from fossil fuels. With their biodegradable properties, they have also recently been applied to the medical field. As poly(3-hydroxybutyrate) produced by wild-type *Ralstonia eutropha* has limitations with regard to its physical properties, it is advantageous to synthesize co- or terpolymers with medium-chain-length monomers. In this study, tung oil, which has antioxidant activity due to its 80% α-eleostearic acid content, was used as a carbon source and terpolymer P(53 mol% 3-hydroxybytyrate-co-2 mol% 3-hydroxyvalerate-co-45 mol% 3-hydroxyhexanoate) with a high proportion of 3-hydroxyhexanoate was produced in *R. eutropha* Re2133/pCB81. To avail the benefits of α-eleostearic acid in the tung oil-based medium, we performed partial harvesting of PHA by using a mild water wash to recover PHA and residual tung oil on the PHA film. This resulted in a film coated with residual tung oil, showing antioxidant activity. Here, we report the first application of tung oil as a substrate for PHA production, introducing a high proportion of hydroxyhexanoate monomer into the terpolymer. Additionally, the residual tung oil was used as an antioxidant coating, resulting in the production of bioactive PHA, expanding the applicability to the medical field.

## 1. Introduction

Tung oil is produced from the seeds of the tung oil tree, which is also called the China wood oil tree or *Vernicia fordii.* As the name suggests, the trees have been cultivated for oil production for thousands of years in China [1]. The global Tung oil market was valued at USD 287 million in 2019 and it is expected to reach USD 344.2 million by the end of 2026, growing at a CAGR of 2.6% during 2021–2026 [2,3].

Almost 80% of tung oil is α-eleostearic acid, (9*Z*,11*E*,13*E*)-octadeca-9,11,13-trienoic acid, which has conjugated double bonds in its backbone. Thus, tung oil has a faster drying time compared to other plant oils of similar iodine value, such as linseed and perilla seed oil. Because of its fast drying time, it is used as a high-quality and high-speed drying oil in the production of paint, varnish, and ink [3,4]. However, over the past few decades, the use of drying oils has declined due to being replaced by alkyd resins and other binders [5]. Therefore, several studies have recently suggested the new application of tung oil [6,7]. Another reason why tung oil is drawing attention is its unique fatty acid composition; α-eleostearic acid has anti-tumor and antioxidant activities [8].

Polyhydroxyalkanoates (PHAs) are renewable and biodegradable biopolymers that bacteria accumulate to store carbon and energy under certain nutrient-limited conditions (e.g., lack of phosphorus, nitrogen, oxygen, or trace elements) or when there is excess carbon supply [6,9,10,11]. PHAs are classified into several types depending on the structure of the repeating units in the polymer [12,13]. They have different properties according to the monomers they contain, such as 3-hydroxybutyrate (3HB), 3-hydroxyvalerate (3HV), and 3-hydroxyhexanoate (3HHx) [14]. Most of the common, naturally occurring PHA granules contain only 3HB, which has poor properties for processing and application as a plastic [15,16]. The copolymer and terpolymer are more similar to petroleum-derived plastics, with lower melting points and higher flexibility [17,18,19]. In addition, PHAs can be applied to various medical fields because they are biodegradable and do not exert any toxic or negative effects on living cells or tissues [20,21]. Further, blends and composites with the resulting products have additional properties, such as anticancer or anti-inflammatory effects [22,23].

*Ralstonia eutropha,* one of the most well-known PHA-producing bacteria, can accumulate PHA at up to 80% of its dry cell weight [24,25,26]. However, the wild-type strain can synthesize only the P(3HB) monomer. In many previous studies, the engineering of *R. eutropha* to produce various PHAs was attempted using different substrates [27,28,29,30,31,32]. In this study, we used the engineered strain *R. eutropha* Re2133/pCB81, which can produce the terpolymer P(3HB-co-3HV-co-3HHx) [33]. It has been reported that this strain can synthesize terpolymers using propionic and butyric acids as a substrate [15,34]. Another feature of this strain is its ability to use oils as a carbon source [29,30,35], and it has been used to produce PHA using plant oils [28,35].

In this study, we selected tung oil as a novel carbon source for PHA production. To produce PHAs (3HB, 3HV, and 3HHx), we used *R. eutropha* Re2133/pCB81, which can produce PHA terpolymers. To evaluate tung oil utilization, we analyzed its composition and used it for the cultivation of the bacterium. Furthermore, for better PHA production, the optimal composition of the production medium was determined; this can govern the PHA content in the microorganisms. Finally, we monitored PHA production over time and compared the antioxidant effects of the PHAs extracted from the strain using this method. This is the first attempt to use tung oil as a microbial substrate to produce bioplastics.

## 2. Materials and Methods

### 2.1. Microorganisms and Culture Conditions

To produce terpolymers, we used the engineered *R. eutropha* strain Re2133/pCB81 (Δ*phaB1*, Δ*phaB2*, Δ*phaB3*, Δ*phaC1*, and *phaC2Ra*-*phaA*-*phaJ1Pa*, containing pBBR1MCS-2 harboring a synthetic PHA operon). The seeding medium was prepared by culturing the strain at 30 °C for 24 h in 5 mL of tryptic soy broth (TSB) supplemented with 200 μg/mL kanamycin and 10 μg/mL gentamicin. Then, the cells were harvested and washed twice with sterilized water, and 0.1% (*v*/*v*) seed culture was used to inoculate 5 mL of M9 minimal medium with 2% (*v*/*v*) filtered tung oil (Liberon, F&H International, Gwangju, Korea) and 0.1% (*w*/*v*) yeast extract. PHA production proceeded at 30 °C for 72 h. The culture was analyzed for growth and PHA production [29].

### 2.2. Tung Oil Composition Analysis Using Gas Chromatography-Mass Spectrometry (GC-MS)

For compositional analysis, the tung oil was diluted with chloroform to obtain a 1% (*v*/*v*) solution. Mild alkaline methanolysis was used for derivatization. For methanolysis, 0.5 mL of methanol, 0.5 mL of toluene, and 1 mL of 0.3 M methanolic-KOH were added to the samples, followed by incubation at 37 °C for 15 min. Then, 2 mL of hexane, 0.3 mL of 1 M-acetic acid, and 2 mL of water were added to the mixture, followed by vortexing to obtain a separated hexane layer on top of the solution. The hexane layer was transferred to a clean glass tube, and the hexane was evaporated with a nitrogen gas evaporator. For resuspension, 1 mL of chloroform was used. Residual water was removed by adding Na_2_SO_4_, and the sample was transferred to a clean borosilicate vial by filtering with a 0.2 μm PVDF filter. Prepared samples were analyzed using a GC-MS column (Calrus 500, Perkin Elmer, Waltham, MA, USA) equipped with a fused silica capillary (Elite-5 ms, 30 m, 0.25 mm, i.d. 0.25 μm film). They were subjected to a linear temperature gradient for fatty acids (held at 120 °C for 5 min, increased at 6 °C/min to 200 °C, increased at 2 °C/min to 220 °C, and then increased at 10 °C/min to 300 °C). The injector port temperature was set at 210 °C. Mass spectra were obtained via electron impact ionization at 70 eV, and scan spectra were obtained within the range of 45–400 m/z. The selected ion mode was used for the detection and fragmentation of major products. The remaining tung oil in the medium was obtained via liquid-liquid extraction using hexane and analyzed as previously reported [36].

### 2.3. Tung Oil Toxicity Testing

Experiments were conducted to investigate the effects of tung oil on the growth of *R. eutropha* 2133/pCB81. The strain was precultured for 24 h at 30 °C in 3 mL of TSB with appropriate antibiotics (10 μg/mL gentamicin and 200 μg/mL kanamycin). The cells were harvested and washed twice with sterilized water and then used to inoculate 5 mL of TSB containing 0.5 or 1% (*v*/*v*) of tung oil. Cell growth was monitored by measuring the optical density of the culture media at 595 nm, starting from an initial value of 0.05 [15].

### 2.4. Optimal Culture Conditions for PHA Production

To find the optimal nitrogen source, *R. eutropha* Re2133/pCB81 was cultured in minimal medium as mentioned above; tung oil was used as the carbon source [29,37]. For nitrogen sources, 0.1% of yeast extract, casein, peptone, tryptone, NH_4_NO_3_, (NH_4_)_2_SO_4_, and NH_4_Cl were evaluated. We selected yeast extract as an optimal nitrogen source, showing the highest yield, and comparative experiments were conducted by varying its concentration. All aforementioned experiments were performed using a shaking incubator at 30 °C (200 rpm) for 72 h.

### 2.5. Batch Fermentation Conditions

To prepare the inoculums for cultivation, *R. eutropha* Re2133/pCB81 was grown overnight in TSB containing appropriate antibiotics, then inoculated to 50 mL of M9 minimal medium flask precultures containing 2% tung oil and 0.2% yeast extract. After overnight cultivation, 0.1% (*v*/*v*) of flask cultured cell was used to inoculate to each fermentor. Each vessel contained 400 mL of optimized PHA production medium. The temperature of the culture was kept at 30 °C. During fermentation, air was supplied at 5 vvm, pH was maintained at 6.8 ± 0.1 through 0.1 N of HCl and NaOH. Stirring was provided by a four-blade Piched impeller at speeds of 400 rpm. Sterile silicone oil LG 50 was used as an antifoam.

### 2.6. PHA Analysis Using GC-MS and ^1^H-NMR

PHA was quantified and characterized using GC-MS according to a previously described method [38]. In brief, the culture broth was centrifuged and washed twice with hexane. The washed cells were transferred into a glass vial for lyophilization, and the dry cell weight was measured. Equal volumes of chloroform and 15% (*v*/*v*) H_2_SO_4_/85% methanol solution (2 mL total volume) were added to the glass vial, and methanolysis was performed for 2 h at 100 °C, followed by cooling to room temperature. A 1-mL aliquot of deionized water was added to the methyl ester solution, which was vortexed for 5 s. The chloroform layer was transferred into a microtube containing crystalline anhydrous Na_2_SO_4_ to remove the residual water. Filtered 1 μL aliquots were injected into the GC-MS (PerkinElmer, Waltham, MA, USA) equipped with triple-axis detector carrying Elite 5 ms column (30 mm length 0.25 mm internal diameter 0.25 film) [39]. For carrier gas, helium was used at 48.3 mLmin^−1^. The temperature of injector was set at 280 °C, while the column and oven were programmed to increase 10 °C for 1 min to 120 °C at 15 °C min^−1^, then hold for 15 min, and increase to 300 at 10 °C min^−1^ hold for 15 min. NIST/EPA/NIH library was used to predict the methylated PHAs. Approximately 20 mg of ^1^H-NMR sample was melted in 1 mL of deuterated chloroform (CDCl_3_) and performed ^1^H-NMR (Bruker Avance III 400 FT-NMR) (data not shown). Peaks of ^1^H-NMR was compared with a previous report [15].

### 2.7. Polymer Extraction and Scanning Electron Microscopy (SEM) of Films

The conventional solvent-cast method was used to prepare the PHA film. To form the PHA film, *R. eutropha* Re2133/pCB81 cells were grown for 72 h in 100 mL of M9 minimal medium containing 2% tung oil or fructose and 0.2% yeast extract with appropriate antibiotics in a 250 mL baffled flask. The culture broth (50 mL) was centrifuged, and the cell pellet was collected. Depending on the type of film, the pellet was washed twice with deionized water or n-hexane and lyophilized. Dried cells were submerged in chloroform (40 mL) and extracted at 60 °C for 16 h. After adding 10 mL of distilled water, the polymer-containing chloroform fraction was collected via centrifugation. The refined solvent was dried in a fume hood at room temperature. When the chloroform evaporated, the PHA film was obtained. The resulting PHA films were used for subsequent analyses.

For SEM analysis, the films were coated with gold and then analyzed using a TM3030Plus tabletop microscope (Hitachi, Tokyo, Japan) [40]. SEM images were obtained at an accelerating voltage of 5 kV.

### 2.8. DPPH Radical-Scavenging Assay

To determine the antioxidant activity of the PHA films, a 1,1-diphenyl-2-picryl-hydrazyl (DPPH) assay was performed to measure the radical-scavenging capacity. To evaluate the antioxidant activity of tung oil, mixtures containing various concentrations of the sample (0.2, 0.4, 0.6, 0.8, and 1%) mixed with 0.5 mL of DPPH (0.01 mM) were prepared in methanol. The mixed samples were incubated in the dark for 30 min, and the DPPH radical-scavenging activity was assessed by measuring the absorbance of the samples at 517 nm using a Gemini XPS and EM Microplate Readers (Molecular devices, San Jose, CA, USA). Ascorbic acid was used as an antioxidant standard. For PHA films, 0.01 g of each polymer was incubated with DPPH solution and analyzed as mentioned above [23,41]. The relative scavenging activity was calculated by comparing the antioxidant activity of polymers to that of ascorbic acid.

### 2.9. Phosphomolybdenum Assay

Antioxidant activity can be investigated based on the reduction of Mo^6+^ to Mo^5+^ subsequently forming phosphomolybdenum complex at acidic pH. Various concentrations of tung oil and 0.01 g of extracted tung oil-coated films were treated with 1 mL of molybdate reagent containing 0.6 M sulfuric acid, 4 mM ammonium molybdate, and 28 mM sodium phosphate in distilled water. The tubes were incubated at 37 °C, and absorbance of the mixture was recorded at 695 nm using a Gemini XPS and EM Microplate Readers (Molecular devices, San Jose, CA, USA). An increase in the absorption of the reaction mixture indicated antioxidant activity. Ascorbic acid was used as a positive control [41,42].

### 2.10. Gel Permeation Chromatography

The molecular weight and molecular mass distribution of PHA were determined using gel permeation chromatography (YL Chromass, Seoul, Korea). For sample preparation, PHA films were dissolved in chloroform and filtered through a 0.2 μm syringe filter (Chromdisc, Seoul, Korea). An HPLC apparatus consisting of a loop injector (Rheodyne 7725i), an isocratic pump with dual heads (YL9112), a column oven (YL9131), columns (Shodex, K-805, 8.0 I.D. × 300 mm, Shodex, K-804, 8.0 I.D. × 300 mm), and a refractive index detector (YL9170) was used. Chloroform was used as the mobile phase (1 mL/min) at 35 °C. Exactly 60 μL of each sample was injected. Data were analyzed using the YL-Clarity software for YL HPLC (YL Chromass, Seoul, Korea). The molecular mass was determined using polystyrene standards ranging from 5000 to 2,000,000 g/mol.

### 2.11. Statistical Analysis

All data are representative of replicate experiments. Statistical significance was determined by one-way ANOVA using MiniTab 18 software at a 95% confidence level. A value of *P* < 0.05 was considered significant.

## 3. Results and Discussion

### 3.1. Utilization of Tung Oil by R. Eutropha 2133/pCB81 for Cell Growth and PHA Production

**Figure 1:**
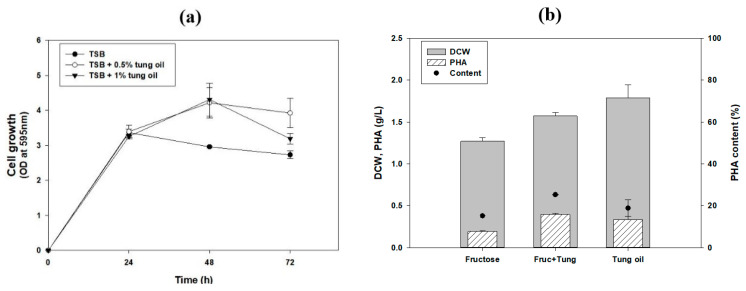

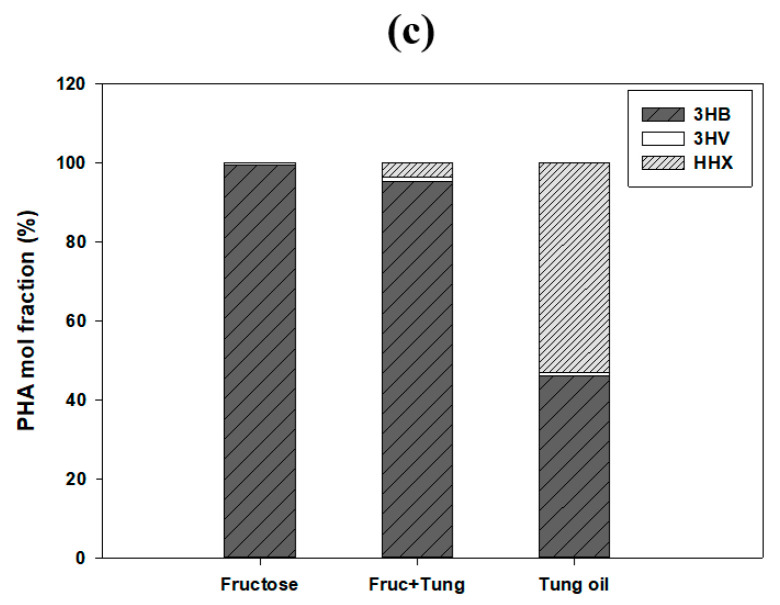
Effect of tung oil on (**a**) cell growth, (**b**) polyhydroxyalkanoate (PHA) production and (**c**) PHA mole fraction in *R. eutropha* 2133/pCB81. Statistical analysis was performed by applying ANOVA with the level of significance at 5%. 3HB, 3-hydroxybutyrate; 3HV, 3-hydroxyvalerate; DCW, dry cell weight; HHx, 3-hydroxyhexanoate; OD, optical density.

α-Eleostearic acid, which makes up more than 80% of tung oil, is known to suppress the growth of cancer cells [8,43,44,45]. It is a powerful biologically active compound that has antioxidant and anticancer properties. Many studies have demonstrated its potential cytotoxicity and apoptosis-inducing activity on human breast cancer cells [8]. According to previous studies, treatment with various polyunsaturated fatty acids can inhibit bacterial cell growth. With regard to *Helicobacter pylori,* which is one of the major causes of peptic ulcers, ω-3 linolenic acid has a high inhibitory effect, and other types of polyunsaturated fatty acids showed similar effects [45]. Pure tung oil is known to be minimally toxic, but it was necessary to determine how it affects cell growth. Therefore, we examined the toxicities of tung oil toward *R. eutropha* 2133/pCB81 (Figure 1a). Filtered tung oil was added to the TSB medium, and the absorbance of samples grown in TSB without the oil was compared. The final concentrations of tung oil used were 0.5 and 1% (*v*/*v*). The growth was similar until 24 h. After 48 h, the optical density of the samples with tung oil was higher than that of those without the oil. Thus, up to 1% tung oil did not inhibit cell growth.

To verify the effects of tung oil on PHA production, the carbon source concentration was fixed at 2% (*v*/*v*) of the total volume. Samples containing 2% fructose, 1% fructose with 1% tung oil, or 2% tung oil in M9 minimal medium were compared for PHA production (Figure 1b,c). Growth and PHA production were higher in samples with tung oil than in those with only fructose. P(99.3 mol*%* 3HB-co-0.7 mol% 3HV) was prepared from samples containing fructose as a sole carbon source. As shown by our data, HHx, as well as 3HB and 3HV, was produced in the medium that contained tung oil. The HHx mole fraction comprised almost 50% of the total PHA when tung oil was used as a single carbon source. These results show that tung oil can be a potential carbon source to produce the PHA terpolymer in *R. eutropha* 2133/pCB81. The HHx content in this PHA, at more than 45 mol%, is the highest to ever be reported with this strain. Previous reports identified that P(HB-co-HHx), containing 42% 3HHx monomer, can be produced in this strain using butyrate [33] and when grown in the presence of palm oil, the same strain can accumulate the polymer at 64% of the dry cell weight (DCW), with up to 23.3 mol% HHx content [28,35]. In the case of coffee waste oil, PHA accumulated at 69% (*w*/*w*) of DCW, consisting of 3HB (78 mol%) and HHx (22 mol%) [29].

### 3.2. Optimization of the Nitrogen Source for PHA Production and Monitoring of PHA Production over Time

**Figure 2:**
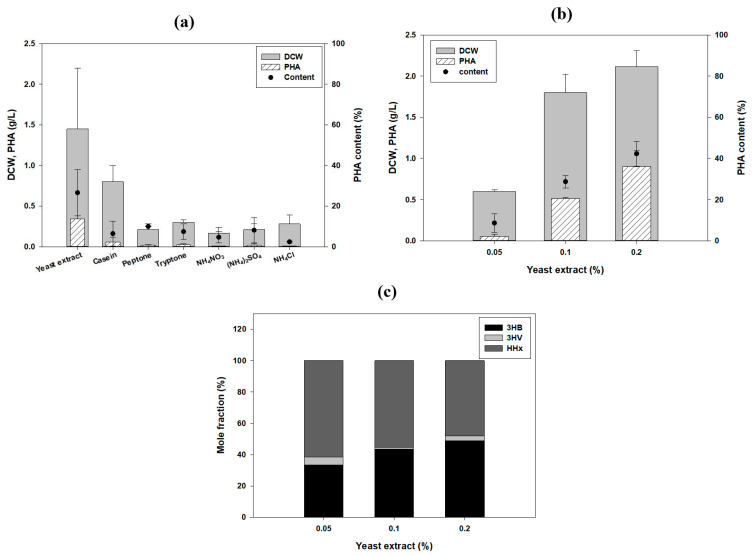
Optimization of the nitrogen source in the polyhydroxyalkanoate (PHA) production medium. (**a**) Cell growth and PHA production were compared in the presence of various nitrogen sources. Effect of the concentration of the yeast extract on (**b**) PHA production and (**c**) PHA mole fraction were obtained. Statistical analysis was carried out by applying ANOVA with the level of significance at 5%. 3HB, 3-hydroxybutyrate; 3HV, 3-hydroxyvalerate; DCW, dry cell weight; HHx, 3-hydroxyhexanoate; OD, optical density.

Cell growth and PHA accumulation can be affected by the nitrogen source used. Therefore, the optimal medium conditions for production of PHA by *R. eutropha* 2133/pCB81 were examined. The concentration of tung oil was fixed at 2% (*v*/*v*). Cell growth and PHA production using various nitrogen sources, including single and complex sources, were compared (Figure 2a). The concentration of the N source was fixed at 0.1% (*v*/*v*) of the final volume. Compared to the other N sources, the yeast extract was the best. By varying the concentration of the yeast extract, we found that 0.2% was the optimum concentration in terms of cell growth (2.22 g dry cell mass/L) and PHA production (1.01 g/L) (Figure 2b). The mole fraction of HHx was up to 61% when 0.05% yeast extract was provided. As the concentration of the yeast extract increased from 0.1 to 0.2%, the total PHA production doubled from 0.506 to 1.013 g/L, while the proportion of HHx decreased from 55 to 48%. Therefore, based on the DCW and PHA contents, 0.2% yeast extract was determined to be the optimum N source (Figure 2c). Although more candidate nitrogen sources might also be possible, we simply selected nitrogen source and focused on the usage of tung oil.

**Figure 3:**
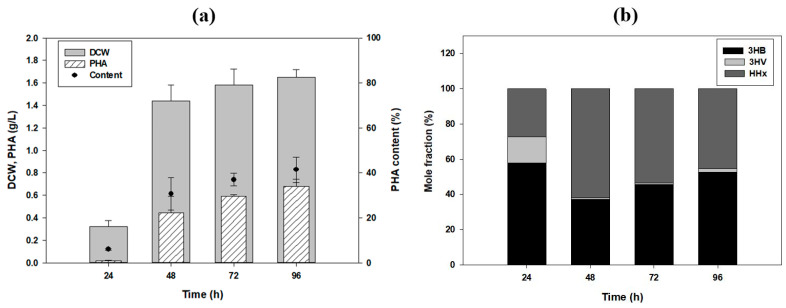
Monitoring of terpolymer production over time using tung oil as the carbon source. (**a**) biomass, polyhydroxyalkanoate (PHA) accumulation; (**b**) PHA monomer composition. 3HB, 3-hydroxybutyrate; 3HV, 3-hydroxyvalerate; DCW, dry cell weight; HHx, 3-hydroxyhexanoate; OD, optical density.

To monitor the PHA production by *R. eutropha* 2133/pCB81 over time, using tung oil as a carbon source, the cells were cultured for 96 h under the optimized conditions (Figure 3a,b). PHA started to accumulate after 24 h, and the maximum amount of PHA (0.68 g/L) and cell biomass (1.65 g/L) were measured at 96 h. The highest HHx mole fraction was 61.9 mol% after 48 h, and the proportion of HHx decreased over time, yielding P (53 mol% 3HB-co-2 mol% 3HV-co-45 mol% HHx) after 96 h. Although the proportion of HHx decreased after 48 h, it was still the highest proportion of HHx fractions reported so far.

The fatty acid composition was calculated based on the peak areas (Table 1). The generally accepted composition of tung oil is 83.3% α-eleostearic acid, 4% oleic acid, 7.5% linoleic acid, and 5.2% palmitic acid. Our GC-MS analysis showed a similar composition, with 83.97% α-eleostearic acid, 7.69% oleic acid, 6.25% linoleic acid, and 2.09% palmitic acid. The tung oil also appeared to contain a small quantity of some other fatty acids, including stearic acid (octadecanoic acid), gondoic acid (11-eicosenoic acid), parinaric acid (octadeca-9,11,13,15-tetraenoate), and arachidic acid (eicosanoic acid) (data not shown). However, we decided to focus on the four main components.

The fatty acid composition of the residual tung oil in the medium was also analyzed. We identified changes in the composition of tung oil (Table 1). The percentage of α-eleostearic acid decreased significantly from 83.97 to 59.21%, which means that the consumption of α-eleostearic acid was greater than that of other fatty acids. The percentage of oleic acid appeared to increase after cultivation. However, we interpreted this to mean that the utilization of α-eleostearic acid was greater than that of oleic acid because the data represent the relative fatty acid proportions, not the absolute amounts.

### 3.3. SEM Images and Antioxidant Activity of PHA Films

**Figure 4:**
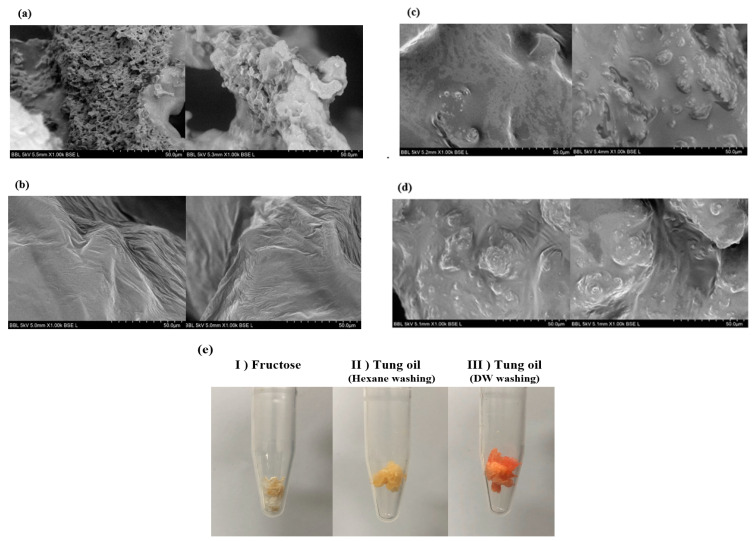
SEM images of the extracted polyhydroxyalkanoate (PHA) films. The surface of the PHA film produced using (**a**) fructose as the sole carbon source. The surface of the PHA film produced using tung oil and (**b**) washed twice with n-hexane, (**c**) washed once with n-hexane; (**d**) washed twice with deionized water; (**e**) Color differences among the films due to remaining tung oil.

SEM analysis of the extracted PHA was conducted. The surface of the PHA produced using fructose as a carbon source, mostly composed of the 3HB monomer, was rough and porous. In contrast, the surface of the P (53 mol% 3HB-co-2 mol% 3HV-co-45 mol% HHx), which was produced using tung oil, had a soft and smooth surface (Figure 4a,b). By performing several hexane washing steps, we removed residual tung oil. However, based on the GC-MS analysis of the residual tung oil after cultivation and the fact that tung oil is used to coat wood and other surfaces, we tried to harvest the tung oil and PHA together as a functional material. As the residual tung oil contained more than 50% α-eleostearic acid, which has anti-oxidative activity, we prepared hexane-washed and water washed-PHA. Using SEM, we observed several lumps on the film washed once with n-hexane (Figure 4c), and many were observed on the surface of the PHA film that was washed with only deionized water. This suggests that a relatively high amount of tung oil remained (Figure 4d). For samples washed twice with hexane, the surface was very smooth, and there were almost no lumps (Figure 4b). The remaining tung oil also caused a difference in the color (Figure 4e) and other properties of the PHA films.

**Figure 5:**
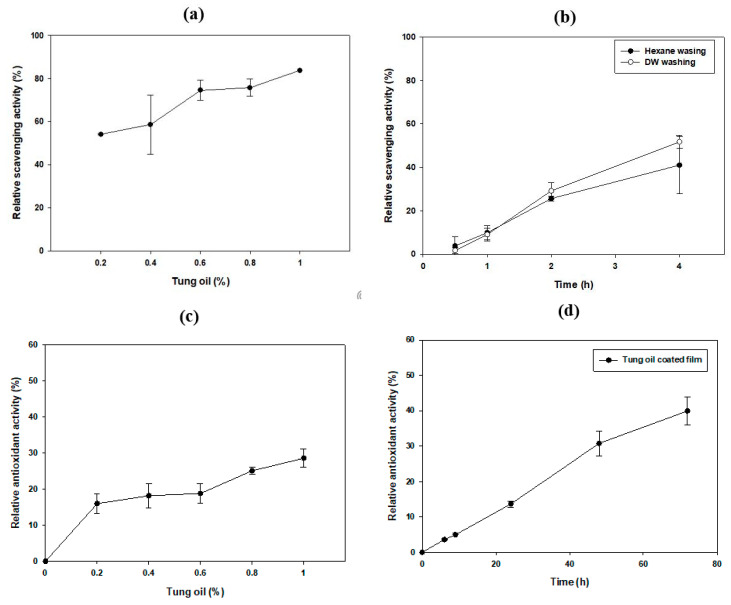
Relative DPPH radical scavenging activity of (**a**) tung oil and (**b**) tung oil-coated films and relative antioxidant activity of (**c**) tung oil and (**d**) tung oil-coated films analyzed by phophomolybdenum assay. Statistical analysis was performed by applying ANOVA with the level of significance at 5%. DW, deionized water.

α-Eleostearic acid is well-known for its antioxidant activity; many studies have shown that it reduces inflammatory reactions and the levels of free radicals in living cells [8]. Scavenging of the stable DPPH radical was used to efficiently identify the antioxidant activity [41]. As shown in Figure 5a, the DPPH radical-scavenging ability of samples increased along with an increase in tung oil concentration in the range of 0–1%. The presence of 1% tung oil showed 83.7% relative antioxidant activity, compared to that of ascorbic acid, which is a potent antioxidant (Figure 5a). Because antioxidant activity was identified in tung oil, the degree of antioxidant activity of the tung oil-coated PHA film was also checked (Figure 5b). Antioxidant activities of the films prepared using tung oil as a carbon source with n-hexane and washing with deionized water were compared; as expected, the films showed 41.02 and 51.71% relative DPPH radical-scavenging activity, respectively, after 4 h. When investigated with the phosphomolybdenum method, we confirmed that antioxidant activity of tung oil increased in a concentration-dependent manner (Figure 5c) [41,42]. Also, with the tung oil-coated PHA film, it showed up to 39.9% antioxidant activity after 96 h, related to ascorbic acid (Figure 5d). Our results show that this simple method of preparing functional bioplastics using tung oil was effective.

There was also a difference in the molecular weight of the synthesized PHA (Table 2). The number-averaged (*M*_n_) and weight-averaged molecular weight (*M*_w_) were analyzed based on polystyrene standards. The molecular weight of the *P*(99.3 mol% 3HB-co-0.7 mol% 3HV) prepared using fructose as a substrate was 360.3 × 10^3^ (PDI: 2.6), and that of *P*(53 mol% 3HB-co-2 mol% 3HV-co-45 mol% HHx) prepared using tung oil was 280.7 × 10^3^ (PDI: 1.8).

### 3.4. Batch Fermentation

**Figure 6:**
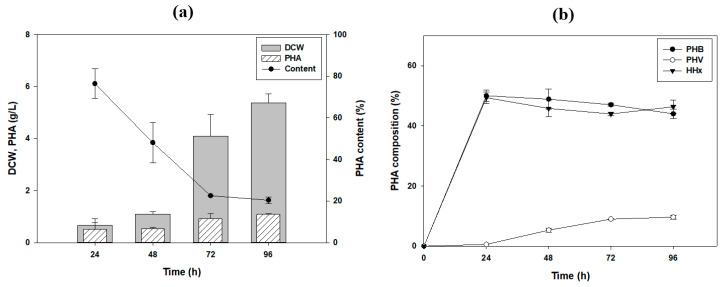
PHA production using tung oil as a carbon source in batch fermentation. (**a**) biomass, polyhydroxyalkanoate (PHA) accumulation, (**b**) PHA monomer composition. 3HB, 3-hydroxybutyrate; 3HV, 3-hydroxyvalerate; DCW, dry cell weight; HHx, 3-hydroxyhexanoate.

In order to show the potential of applying tung oil to PHA production, we conducted batch fermentation. In the batch fermentation, the strain produced 5.37 g/L and of DCW and 1.09 g/L of PHA production, showing 20.4% of PHA content after 96 h (Figure 6a). Considering the mole fraction of the PHA, high HHx containing terpolymer P (44 mol% 3HB-co-10 mol% 3HV-co-46 mol% HHx) was produced. We found that a high proportion of HHx was maintained in the scale-up process. (Figure 6b). According to a previous paper using palm oil as a carbon source in the same strain, it showed 2.9 g/L of DCW with 67% PHA content containing 23.3 mol% HHx at 72 h [28]. In another study, the researchers compared PHA monomer composition after producing PHA with different plant oils. The 3HHx mole fraction with soybean, corn, olive oil cultures were 17.8, 17.3, and 17.2%, respectively. Compared with previous other plant oils, it showed relatively low PHA content with different compositions. As this is an initial application of fermentation, titer would be increased by optimization of fermentation in the near future.

## 4. Conclusions

PHA is a new candidate biopolymer to replace petrochemical plastics. However, the 3HB homopolymer is brittle, breakable, and inflexible as a thermoplastic [28]. Thus, several previous studies have attempted to produce PHA, including long-chain monomers, through genetic engineering of different microorganisms using various substrates [27,29,30,33,39].

In this study, we reveal that tung oil, obtained from the nut seed of the tung tree, can be applied as a substrate for *R. eutropha* 2133/pCB81 to produce P(3HB-co-3HV-co-HHx) without inhibiting cell growth. This method yielded P(53 mol% 3HB-co-2 mol% 3HV-co-45 mol% HHx) with a molecular weight of 280.7 × 10^3^ (PDI: 1.8). This method resulted in the production of PHA with the highest HHx mol% achieved so far from an oil-based method with engineered *Ralstonia*. In addition, the PHA film was coated with the remaining tung oil through a mild water washing step. The oil coating the surface of the PHA films was identified through SEM analysis, and the expected antioxidant activity was verified.

Coating the surface of PHA with an antioxidant provides a potential application of bioplastic to medical materials such as sutures, bio-implants, or drug carriers [29]. The application of tung oil, which is mainly used for wood finishing, to bio-industry is a fresh idea that not only expands the possible uses of tung oil but also greatly affects the characteristics of PHA. Considering previous approaches, our approach is unique not only in producing a 100% bio-based and bioactive bioplastic using a simple harvesting method, but also in providing a novel use of tung oil. Furthermore, by conducting batch fermentation, titer was easily increased in simple scale-up cultivation showing that tung oil is a suitable carbon source for PHA production. Optimization of Fermentation with tung oil will improve the use of tung oil and production of tung oil-based PHA production in the near future.

## Figures and Tables

**Table 1:** Comparison of fatty acid composition of tung oil before and after fermentation.

Fatty Acid Composition (%)			
Fatty Acid		Before	After
C16:0	Palmitic acid	2.09	1.83
C18:1	Oleic acid	7.69	3.6
C18:2	Linoleic acid	6.25	35.36
C18:3	α-eleostearic acid	83.97	59.21
Sum		100	100

**Table 2:** Molecular weights and polydispersities of PHA films.

Polymer	*M_n_* (10^3^)	*M_w_* (10^3^)	PDI
*P*(99.3 mol% 3HB-co-0.7 mol% 3HV)	134.3±2.3	360.2±3.8	2.68
*P*(53 mol% 3HB-co-2 mol% 3HV-co-45 mol% HHx)	156.6±25.3	280.8±20.8	1.82

## Data Availability

The data presented in this study are available on request from the corresponding author.
